# Supplementary material for: Synovial Fluid and Serum MicroRNA Signatures in Equine Osteoarthritis
Source: Int J Mol Sci. 2025 Nov 19;26(22):11190. doi: 10.3390/ijms262211190 (PMC12652959; doi:10.3390/ijms262211190)
Supplement: Supplementary file 1 [file ijms-26-11190-s001.zip › ijms-3960794-supplementary.pdf]

Supplementary S1

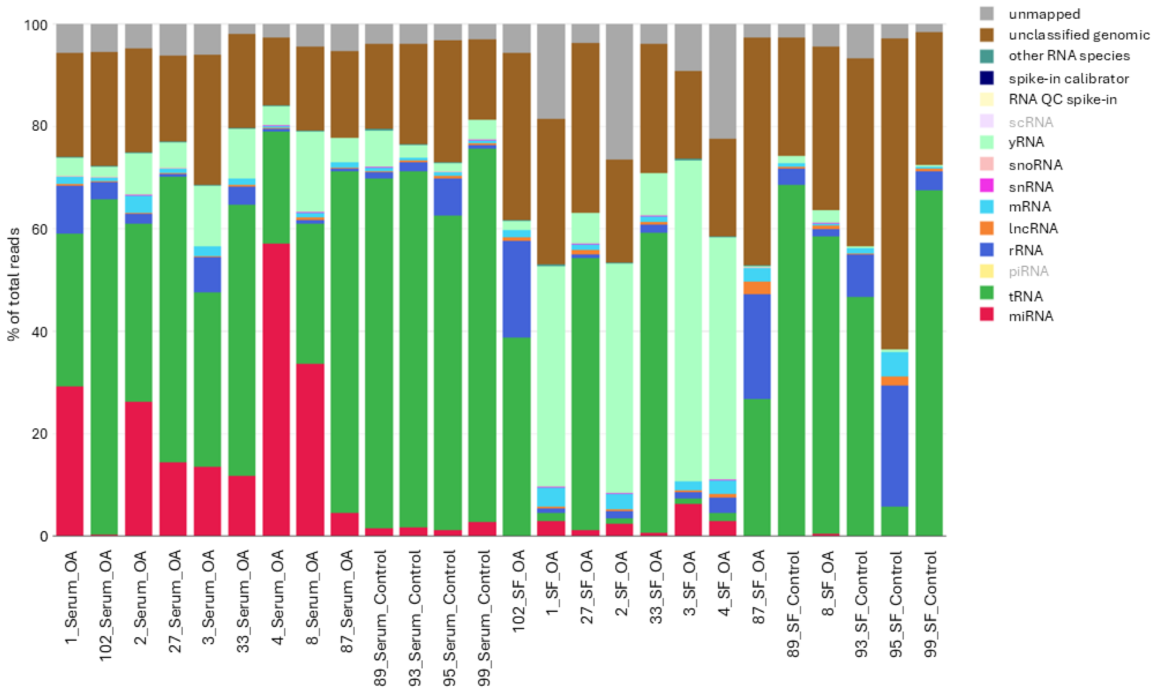

Figure S1. Relative reads composition of all serum and synovial fluid samples. Labels with lower opacity (scRNA and piRNA) correspond to RNA types that were not identified in this dataset. The different bars represent the different samples, with sample ID and group indicated below each bar. lncRNA, long non-coding RNA; mRNA, messenger RNA; miRNA, microRNA; piRNA, piwi-interfering RNA; QC, quality control; rRNA, ribosomal RNA; scRNA, small conditional RNA; snRNA, small nuclear RNA; snoRNA, small nucleolar RNA; tRNA, transfer RNA.

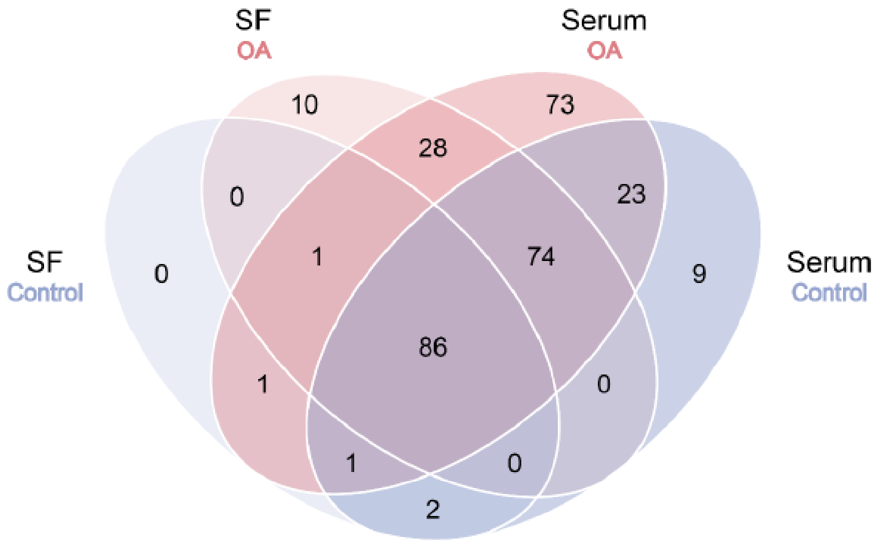

Figure S2. Venn diagram of the distribution of miRNA molecules, stratified by sample type and by group. miRNA, microRNA; OA, osteoarthritis; SF, synovial fluid.

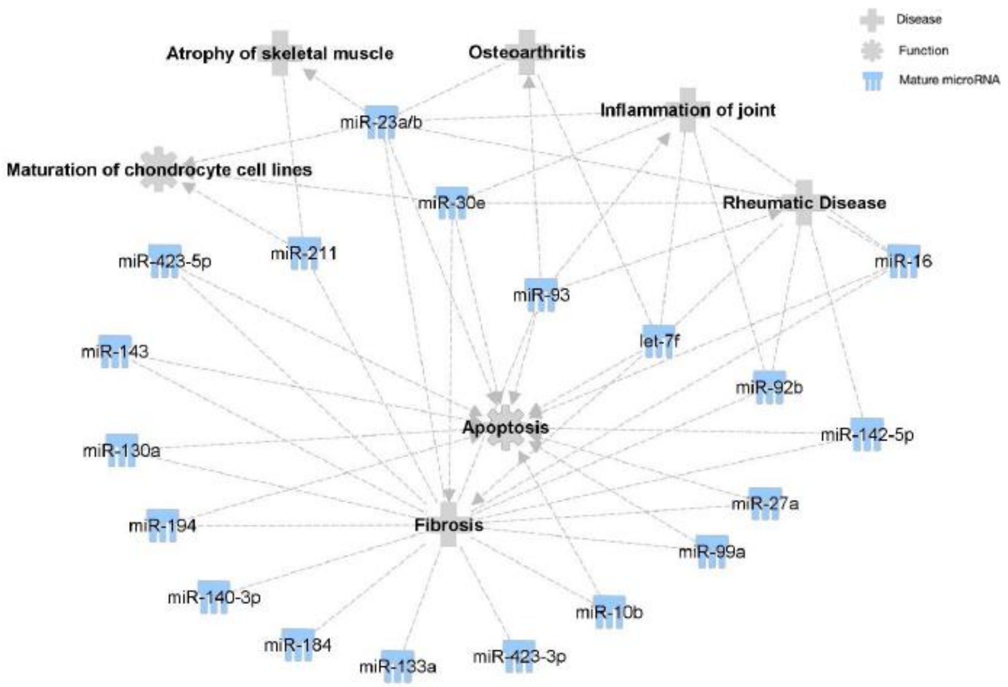

Figure S3. Selected list of diseases and functions predicted to be associated to the differentially expressed miRNAs in either serum or synovial fluid. miRNAs are represented in blue and predicted cellular functions and diseases are represented in grey. Dashed lines represent the relationships between the molecules and predicted functions. Image made in IPA. IPA, Ingenuity Pathway Analysis; miRNA, microRNA.

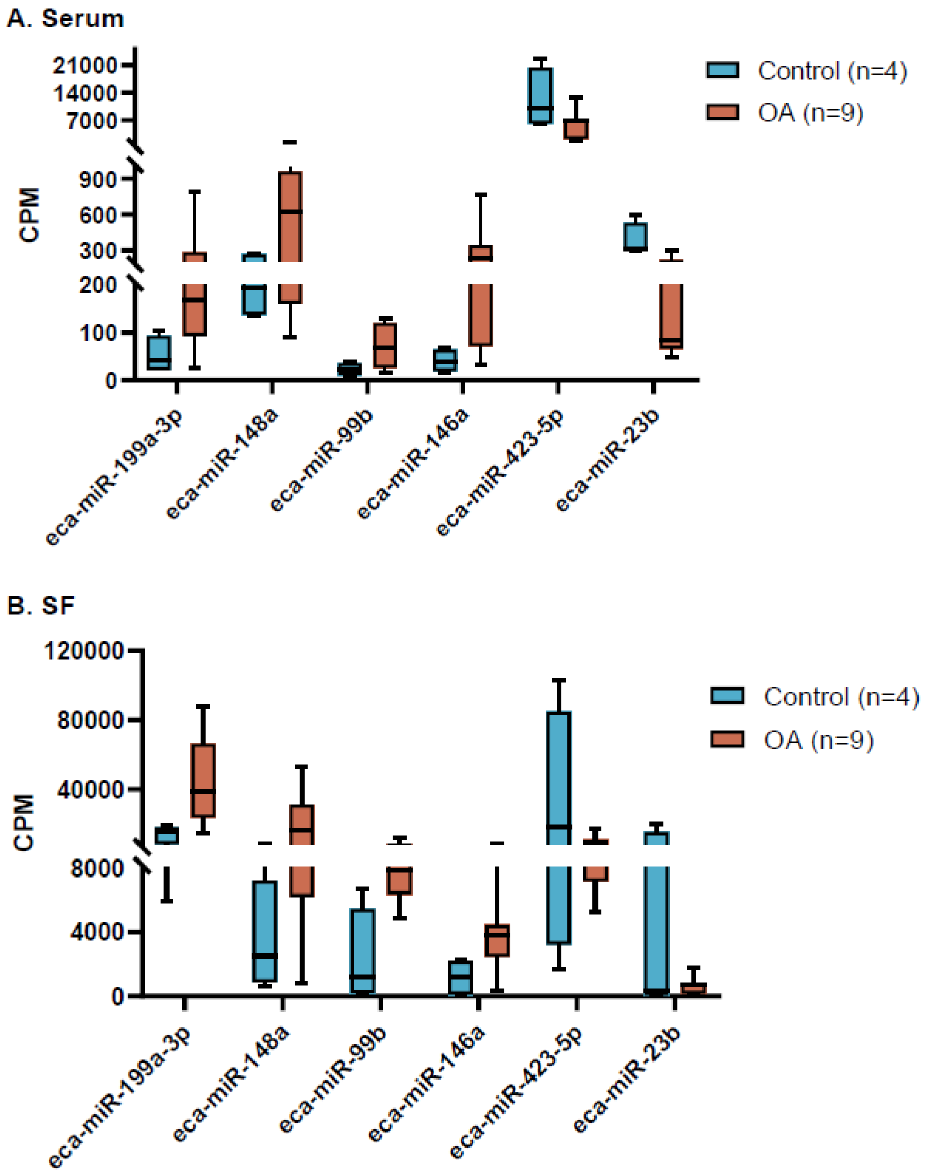

Figure S4. Expression (CPM) of selected miRNAs in the (A) serum and (B) synovial fluid sequencing cohorts. Box plots show the interquartile data range as box boundaries, with error bars showing the minimum and maximum data range, and the horizontal line showing the median. Images were made using GraphPad Prism version 8.0 for Windows. CPM, counts per million; OA, osteoarthritis.

Top Canonical Pathways

Name	p-value	Overlap
Ribonucleotide Reductase Signaling Pathway	2.96E-02	1.2 % 2/170
Role of Chondrocytes in Rheumatoid Arthritis Signaling Pathway	2.00E-01	0.7 % 1/141
Inhibition of ARE-Mediated mRNA Degradation Pathway	2.28E-01	0.6 % 1/163
Cachexia Signaling Pathway	4.44E-01	0.3 % 1/368
Neutrophil Extracellular Trap Signaling Pathway	4.71E-01	0.3 % 1/399

Top Diseases and Bio Functions

Name	p-value range	# Molecules
Inflammatory Disease	4.79E-02 - 1.30E-25	28
Inflammatory Response	4.49E-02 - 1.30E-25	25
Organismal Injury and Abnormalities	4.94E-02 - 1.30E-25	37
Renal and Urological Disease	2.61E-04 - 1.30E-25	19
Neurological Disease	4.94E-02 - 7.18E-23	27

Molecular and Cellular Functions

Name	p-value range	# Molecules
Cellular Movement	3.29E-02 - 8.91E-13	25
Cellular Development	4.94E-02 - 8.94E-12	26
Cellular Growth and Proliferation	4.94E-02 - 8.94E-12	25
Cell Cycle	4.64E-02 - 3.80E-08	8
Cell Death and Survival	4.49E-02 - 3.59E-07	22

Top Diseases and Bio Functions

Name	p-value range	# Molecules
Inflammatory Disease	4.79E-02 - 1.30E-25	28
Inflammatory Response	4.49E-02 - 1.30E-25	25
Organismal Injury and Abnormalities	4.94E-02 - 1.30E-25	37
Renal and Urological Disease	2.61E-04 - 1.30E-25	19
Neurological Disease	4.94E-02 - 7.18E-23	27

Molecular and Cellular Functions

Name	p-value range	# Molecules
Cellular Movement	3.29E-02 - 8.91E-13	25
Cellular Development	4.94E-02 - 8.94E-12	26
Cellular Growth and Proliferation	4.94E-02 - 8.94E-12	25
Cell Cycle	4.64E-02 - 3.80E-08	8
Cell Death and Survival	4.49E-02 - 3.59E-07	22

Figure S5. Summary of IPA Core Analysis performed on differentially expressed miRNAs in serum and synovial fluid. Results were filtered for experimentally observed relationships. IPA, Ingenuity Pathway Analysis.

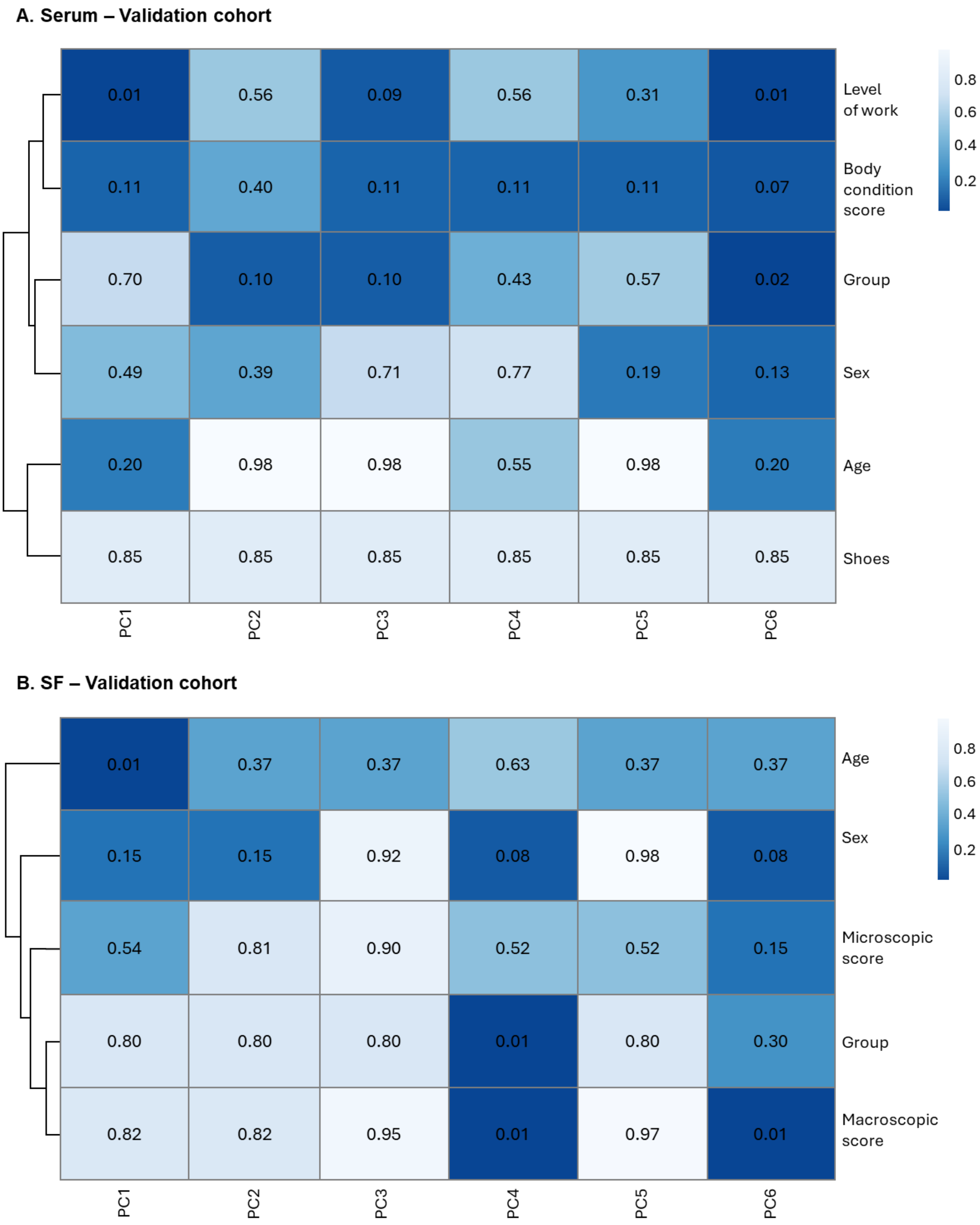

Figure S6. Heatmap of the association of clinical variables of horses in the (A) serum and (B) SF validation cohorts, with the six PCs of miRNA expression. PCAs were performed on normalized and scaled miRNA data obtained by RT-qPCR; these included eca-miR-199a-3p, eca-miR-148a, eca-miR-99b, eca-miR-146a, eca-miR-423-5p and eca-miR-23b. Association between PCs and clinical variables was calculated using One-Way ANOVA for categorical variables, and linear models for numerical variables; results were FDR-adjusted using Benjamini-Hochberg correction. Key on the right denotes FDR-adjusted p-values. Statistical significance was set at FDR p-value<0.05. FDR, false discovery rate; ; miRNA, microRNA; PCA, principal component analysis; PC, principal component; RT-qPCR, reverse transcription quantitative polymerase chain reaction; SF, synovial fluid.

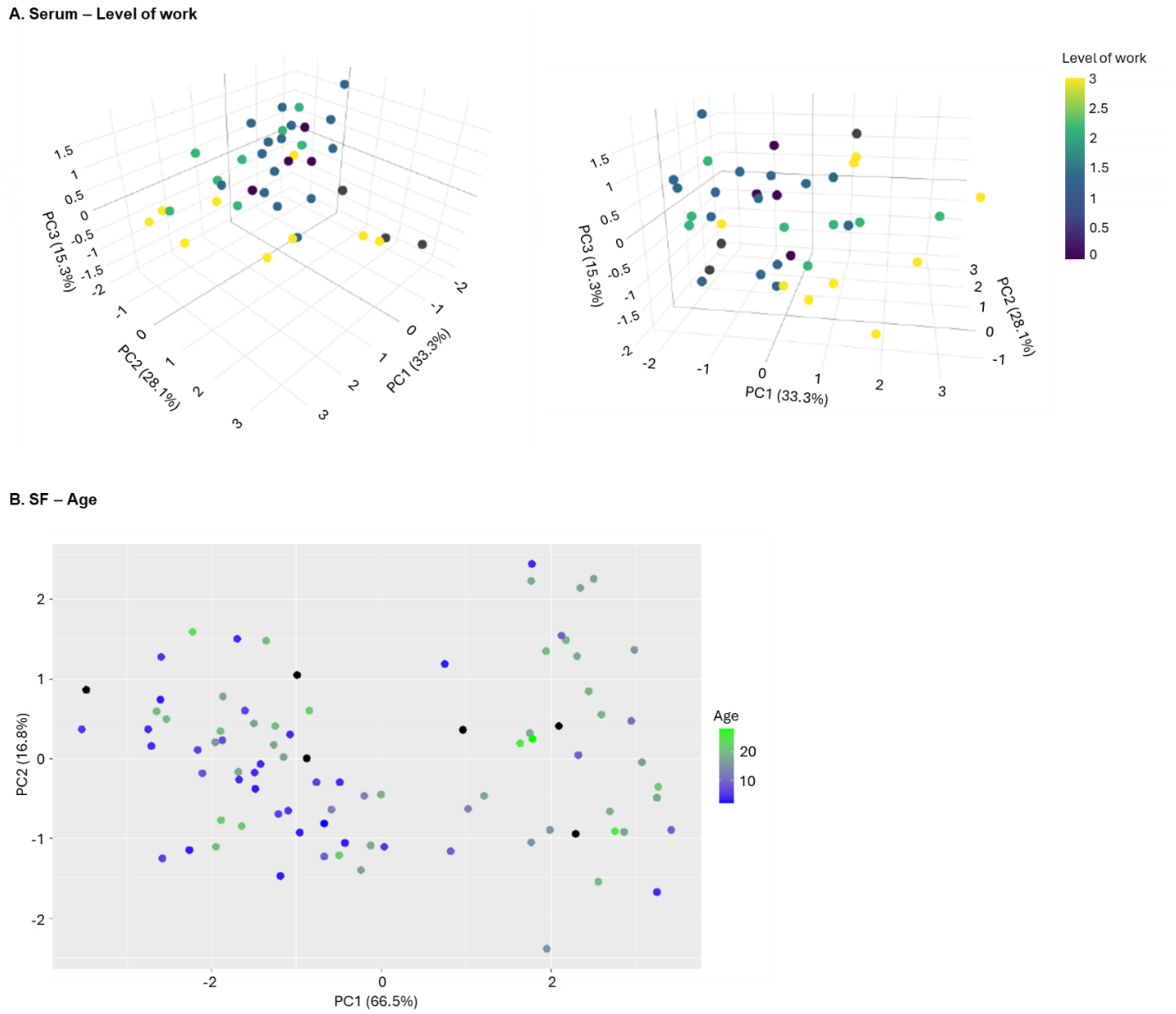

Figure S7. PCA plot of normalized miRNA expression in the (A) serum validation cohort, stratified by level or work; and in the (B) SF validation cohort, stratified by age. miRNA data was obtained using RT-qPCR, normalized and scaled; PCs were calculated using singular value decomposition. microRNA; PC, principal component; PCA, principal component analysis; RT-qPCR, reverse transcription quantitative polymerase chain reaction; SF, synovial fluid.

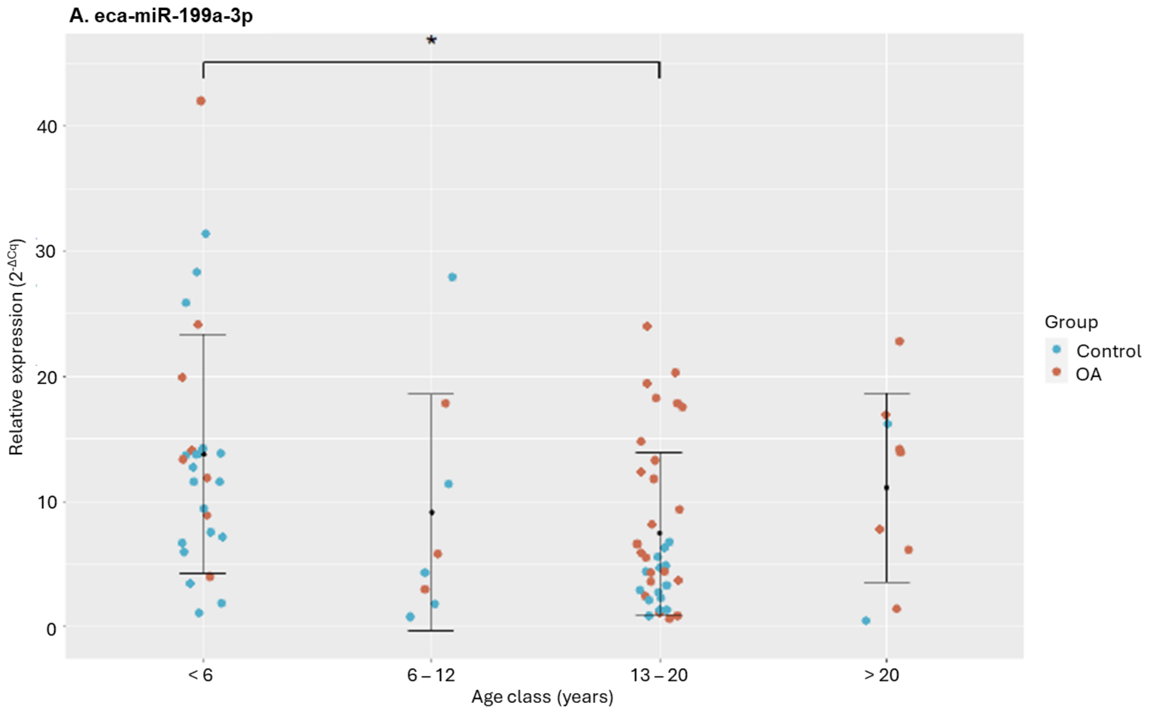

A. eca-miR-199a-3p
*
Relative expression (2-ΔCq)
40
30
20
10
0
< 6
6 – 12
13 – 20
> 20
Age class (years)
Group
Control
OA

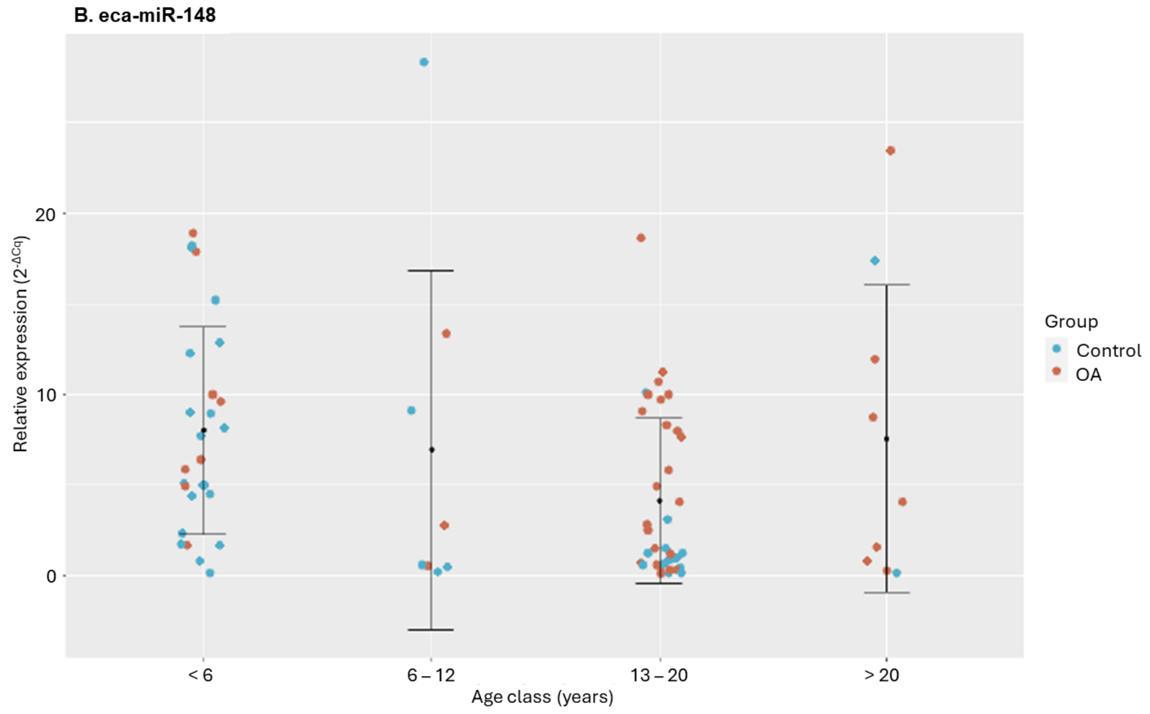

B. eca-miR-148
Relative expression (2-ΔCq)
20
10
0
< 6
6 – 12
13 – 20
> 20
Age class (years)
Group
Control
OA

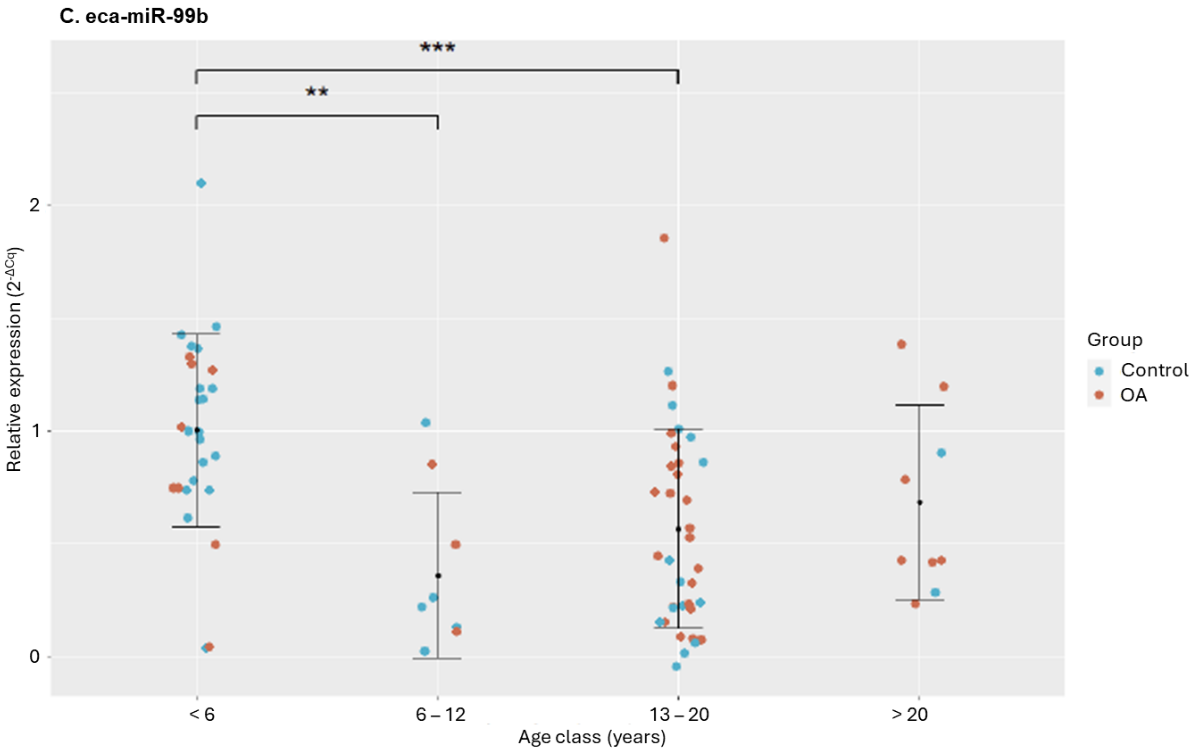
C. eca-miR-99b
***
**
2
1
0
Relative expression (2-ΔCq)
< 6
6 – 12
13 – 20
> 20
Age class (years)
Group
Control
OA

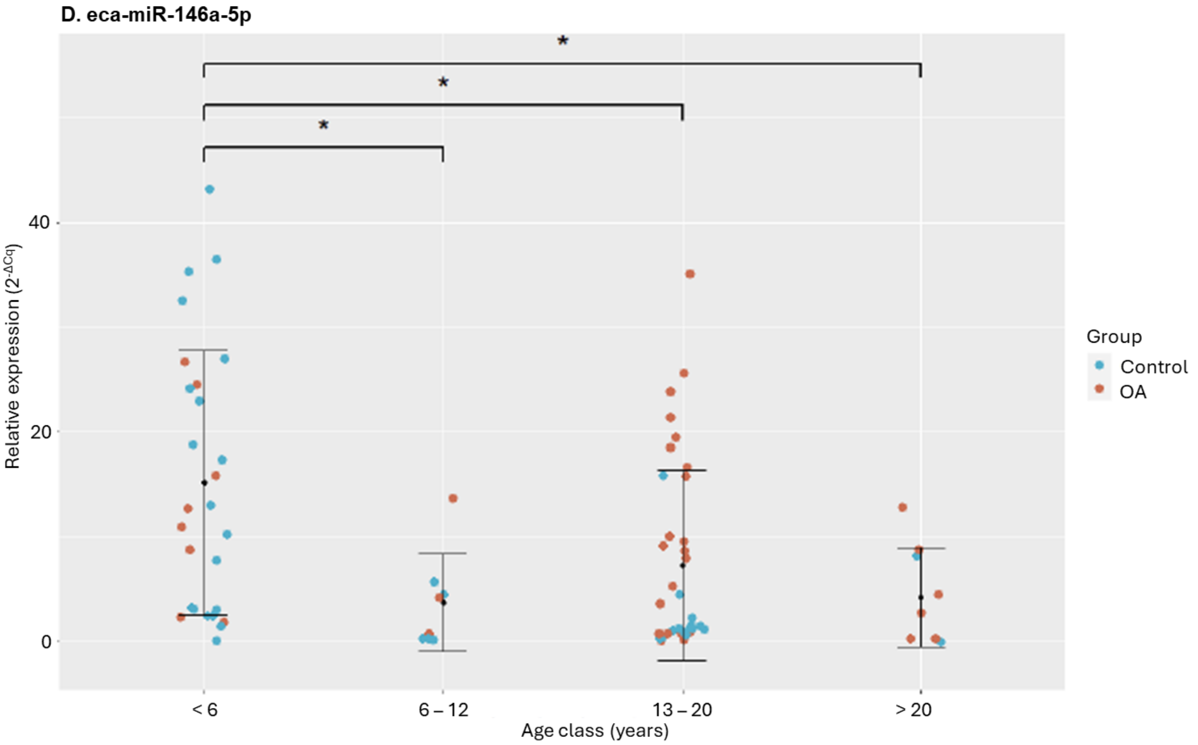
D. eca-miR-146a-5p
*
*
*
40
20
0
Relative expression (2-ΔCq)
< 6
6 – 12
13 – 20
> 20
Age class (years)
Group
Control
OA

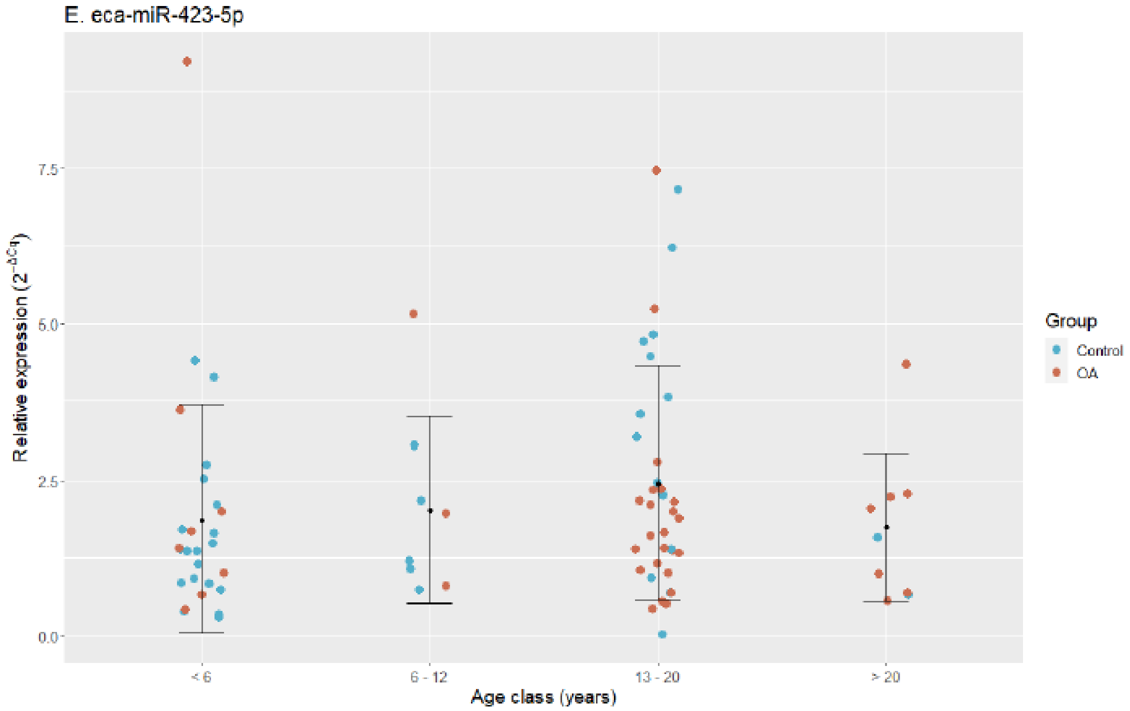
E. eca-miR-423-5p
Relative expression ($2^{-\Delta Cq}$)
7.5
5.0
2.5
0.0
< 6
6 - 12
13 - 20
> 20
Age class (years)
Group
Control
OA

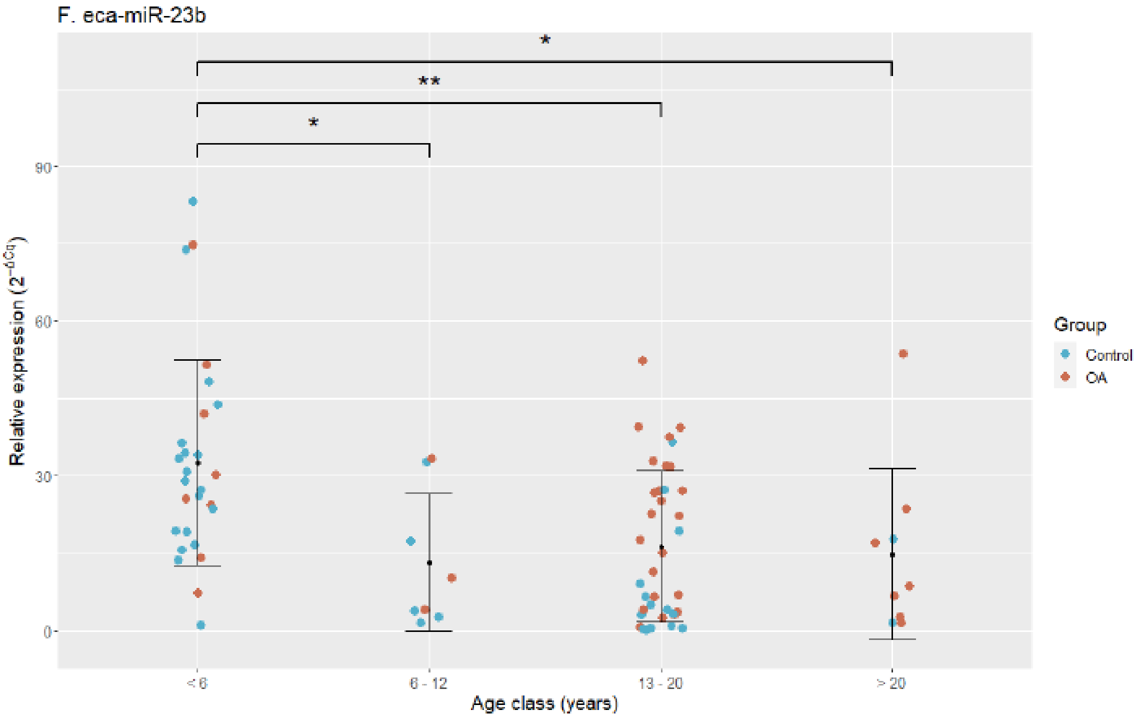
F. eca-miR-23b
*
**
*
Relative expression ($2^{-\Delta Cq}$)
90
60
30
0
< 6
6 - 12
13 - 20
> 20
Age class (years)
Group
Control
OA

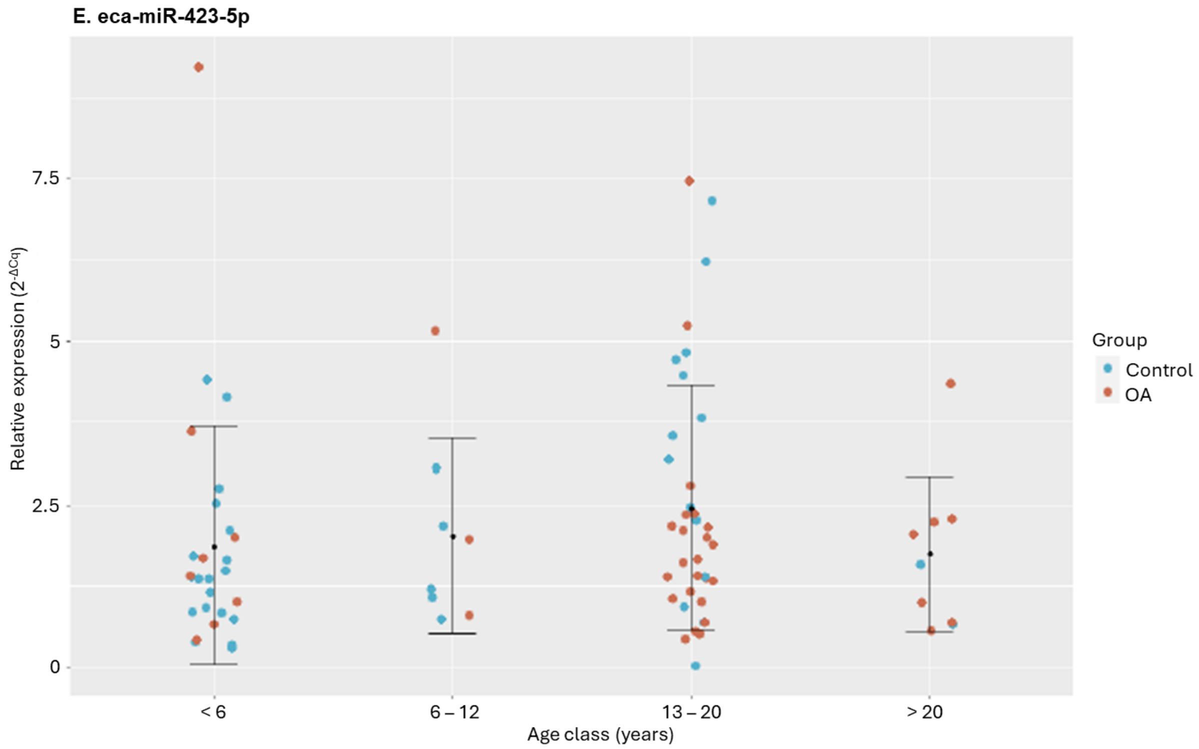

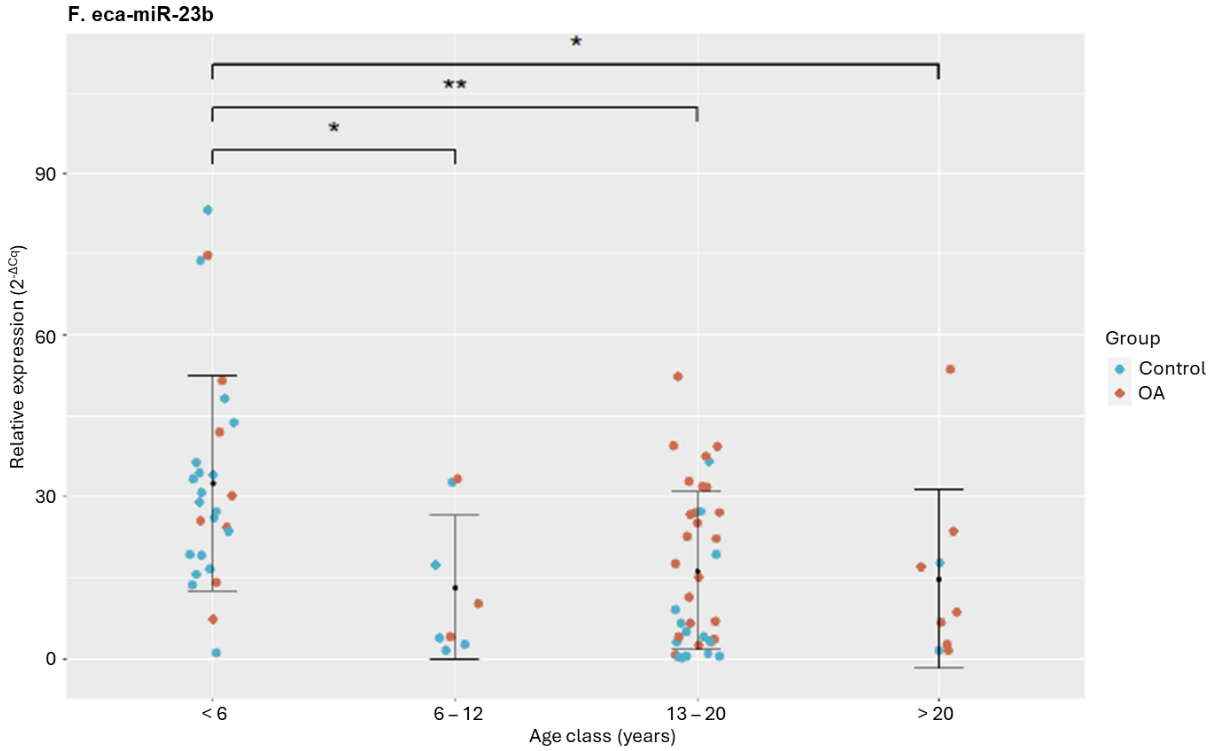

Figure S8. Plots showing relative expression of (A) eca-miR-199a-3p, (B) eca-miR-148a, (C) eca-miR-99b-5p, (D) eca-miR-146a-5p, (E) eca-miR-423-5p and (F) eca-miR-23b in SF, by age class and group. miRNA expression data were obtained by RT-qPCR and normalized to the geometric mean of the tested reference genes. Black circles represent the mean, and error bars represent ± SD. Statistical analyses were performed using GraphPad Prism v8.0, and plots were generated in R. *, FDR p-value<0.05; **, FDR p-value<0.01; ***, FDR p-value<0.001. Cq, quantification cycle; miRNA, microRNA; OA, osteoarthritis; RT-qPCR; reverse transcription quantitative polymerase chain reaction; SD, standard deviation; SF, synovial fluid.

Table S1. Qiagen primer assays used for qPCR detection of miRNAs.

Target	miRCURY LNA miRNA PCR Assay	GeneGlobe ID
eca-miR-199a-3p	hsa-miR-199a-3p	YP00204536
eca-miR-199b-3p	hsa-miR-199a-3p	YP00204536
eca-miR-423-5p	hsa-miR-423-5p	YP00205624
eca-miR-148a	hsa-miR-148a-3p	YP00205867
eca-miR-23b	hsa-miR-23b-3p	YP00204790
eca-miR-99b	hsa-miR-99b-5p	YP00205983
eca-miR-146a	hsa-miR-146a-5p	YP00204688
eca-miR-181b	hsa-miR-181b-5p	YP00204530
eca-miR-107b	hsa-miR-107	YP00204468
eca-miR-486-5p	hsa-miR-486-5p	YP00204001
eca-miR-423-3p	hsa-miR-423-3p	YP00204488
eca-miR-191a	hsa-miR-191-5p	YP00204306

miRNAs, microRNAs; qPCR, quantitative polymerase chain reaction.

Supplementary S2

Selection of candidate reference genes

Five miRNAs were selected to be tested as reference genes: two were manually selected, two were selected using NormFinder [1,2], and one was a synthetic RNA spike-in (exogenous control). Manual selection was based on miRNA stability across all study samples, considering only molecules that were not differentially expressed (p>0.05) with a logFC close to 0. For selection using NormFinder, miRNAs were first filtered based on a minimum of 15 RPM per sample, and the software was used for SF and serum samples separately. The two most stable genes selected by NormFinder for each sample type were tested as reference genes.

Normalization strategies for RT-qPCR and comparison of performance

Four different normalization methods were compared to find the most robust strategy for analysis of miRNA data. These were normalizing data to a single most stable reference gene that was manually selected; normalizing data to a single most stable reference gene or combination of genes as ranked by NormFinder algorithm; normalizing data to the geometric mean of all tested reference genes; and normalizing data to an exogenous oligonucleotide (spike-in). To assess the performance of these normalization strategies, data for all tested miRNAs (including candidate reference miRNAs and differentially expressed miRNAs) were normalized using the $2^{-\Delta Cq}$ method [3] and compared with the log transformed raw (not normalized) Cq data (2^{-rawCq}). The coefficient of variation (CV) was calculated for each individual miRNA after normalization, and CVs for all miRNAs were averaged for each of the normalization techniques. Average CV was used as a normalization performance measure, with lower CVs representing a better removal of experimentally induced noise. The cumulative distribution of the individual CV values was plotted for both raw and normalized data. Samples for which Cq>39 were removed from further analyses.

References:

1. Andersen, C.L.; Jensen, J.L.; Ørntoft, T.F. Normalization of Real-Time Quantitative Reverse Transcription-PCR Data: A Model-Based Variance Estimation Approach to Identify Genes Suited for Normalization, Applied to Bladder and Colon Cancer Data Sets. *Cancer Res* **2004**, *64*, 5245–5250, doi: 10.1158/0008-5472.CAN-04-0496.
2. Tonge, D.P.; Gant, T.W. Evidence Based Housekeeping Gene Selection for MicroRNA-Sequencing (MiRNA-Seq) Studies. *Toxicol Res (Camb)* **2013**, *2*, 328–334, doi: 10.1039/c3tx50034a.
3. Livak, K.J.; Schmittgen, T.D. Analysis of Relative Gene Expression Data Using Real-Time Quantitative PCR and the 2−ΔΔCT Method. *Methods* **2001**, *25*, 402–408, doi: 10.1006/meth.2001.1262
